# Characterization of Responses to Lenvatinib plus Pembrolizumab in Patients with Advanced Renal Cell Carcinoma at the Final Prespecified Survival Analysis of the Phase 3 CLEAR Study

**DOI:** 10.1016/j.eururo.2024.03.015

**Published:** 2024-04-06

**Authors:** Robert J. Motzer, Toni K. Choueiri, Thomas Hutson, Sun Young Rha, Javier Puente, Aly-Khan A. Lalani, Eric Winquist, Masatoshi Eto, Naveen S. Basappa, Nizar M. Tannir, Ulka Vaishampayan, Georg A. Bjarnason, Stéphane Oudard, Viktor Grünwald, Joseph Burgents, Ran Xie, Jodi McKenzie, Thomas Powles

**Affiliations:** a Memorial Sloan Kettering Cancer Center, New York, NY, USA; b Dana-Farber Cancer Institute, Boston, MA, USA; c Texas Oncology, Dallas, TX, USA; d Yonsei Cancer Center, Yonsei University Health System, Seoul, South Korea; e Hospital Clinico San Carlos, Madrid, Spain; f Juravinski Cancer Centre, McMaster University, Hamilton, Canada; g University of Western Ontario, London, Canada; h Kyushu University, Fukuoka, Japan; i Cross Cancer Institute, University of Alberta, Edmonton, Canada; j The University of Texas MD Anderson Cancer Center, Houston, TX, USA; k Rogel Cancer Center, University of Michigan, Ann Arbor, MI, USA; l Sunnybrook Odette Cancer Centre, University of Toronto, Toronto, Canada; m Georges Pompidou European Hospital, University Paris Cité, Paris, France; n University Hospital Essen, Essen, Germany; o Merck & Co., Inc., Rahway, NJ, USA; p Eisai Inc., Nutley, NJ, USA; q Barts Cancer Institute and the Royal Free Hospital, Queen Mary University of London, London, UK

**Keywords:** Renal cell carcinoma, Lenvatinib plus pembrolizumab, Lenvatinib, Pembrolizumab

## Abstract

In the phase 3 CLEAR trial, lenvatinib plus pembrolizumab (L + P) showed superior efficacy versus sunitinib in treatment-naïve patients with advanced renal cell carcinoma (aRCC). The combination treatment was associated with a robust objective response rate of 71%. Here we report tumor responses for patients in the L + P arm in CLEAR, with median follow-up of ~4 yr at the final prespecified overall survival (OS) analysis. Tumor responses were assessed by independent review using Response Evaluation Criteria in Solid Tumors v1.1. Patients with a complete response (CR; *n* = 65), partial response (PR) with maximum tumor shrinkage ≥75% (near-CR; *n* = 59), or PR with maximum tumor shrinkage <75% (other PR; *n* = 129), were characterized in terms of their baseline characteristics. The median duration of response was 43.7 mo (95% confidence interval [CI] 39.2–not estimable) for the CR group, 30.5 mo (95% CI 22.4–not estimable) for the near-CR group, and 17.2 mo (95% CI 12.5–21.4) for the other PR group. The 36-mo OS rates were consistently high in the CR (97%), near-CR (86%), and other PR (62%) groups. Robust objective response rates were observed across International Metastatic RCC Database Consortium favorable-risk (69%, 95% CI 60–78%), intermediate-risk (73%, 95% CI 67–79%), and poor-risk (70%, 95% CI 54–85%) subgroups. The robust response to L + P supports this combination as a standard-of-care first-line treatment for patients with aRCC.

## Patient summary

The CLEAR trial enrolled patients with advanced kidney cancer who had not previously received any treatment for their cancer. Here we report results for tumor shrinkage observed in the group that received lenvatinib plus pembrolizumab combination treatment during the trial. Shrinkage of target tumors with this combination was long-lasting and was observed in patients irrespective of their disease severity.

In the open-label, multicenter, randomized phase 3 CLEAR trial of patients with advanced renal cell carcinoma (aRCC), lenvatinib plus pembrolizumab (L + P) showed significant/clinically meaningful improvements in progression-free survival, overall survival (OS), and the objective response rate (ORR) versus sunitinib [1]. These benefits were sustained at the prespecified final OS analysis at a median survival follow-up of 49.8 mo in the L + P arm [2]. Median follow-up duration for the 174 survivors in the L + P arm at this final OS analysis time point was 49.9 mo (95% confidence interval [CI] 48.9–50.5%). Notable outcome results associated with this combination regimen included the high ORR and the duration of response (DOR); the ORR relative risk was 1.94 (95% CI 1.67–2.26) and the hazard ratio (HR) for DOR was 0.57 (95% CI 0.43–0.76) versus sunitinib [2].

Here we report characteristics of responses in the L + P arm of CLEAR at the final OS analysis, with median survival follow-up duration of ~4 yr.

Full details of the trial have already been reported [1,2]. Treatment-naïve patients (*n* = 355) who had aRCC with a clear-cell component were randomly assigned to receive lenvatinib 20 mg orally once per day plus pembrolizumab 200 mg intravenously once every 3 wk [1]. Of these 355 patients, 16 were not evaluable or had an unknown response, 253 achieved an objective response, 67 had stable disease, and 19 had progressive disease as the best response. Response outcome data presented here correspond to the data cutoff date for the final prespecified OS analysis (July 31, 2022), with 23 mo of additional follow-up beyond the primary analysis [1].

Tumor responses were assessed by independent imaging review using Response Evaluation Criteria in Solid Tumors v1.1. The baseline characteristics for patients with a complete response (CR), near-CR (defined as partial response [PR] with maximum tumor shrinkage of ≥75% from baseline), or other PR (defined as PR with maximum tumor shrinkage <75%) were further assessed. Median OS and quartiles for DOR among responders were calculated using the Kaplan-Meier method; 95% CIs were estimated using a generalized Brookmeyer and Crowley method. OS rates and 95% CIs were calculated using the Kaplan-Meier product-limit method and Greenwood formula. A waterfall plot for patients with both baseline and at least one post-baseline target lesion assessment was generated. The protocol and related documents were approved by institutional review boards or independent ethics committees. All patients provided written informed consent.

**Fig. 1 –:**
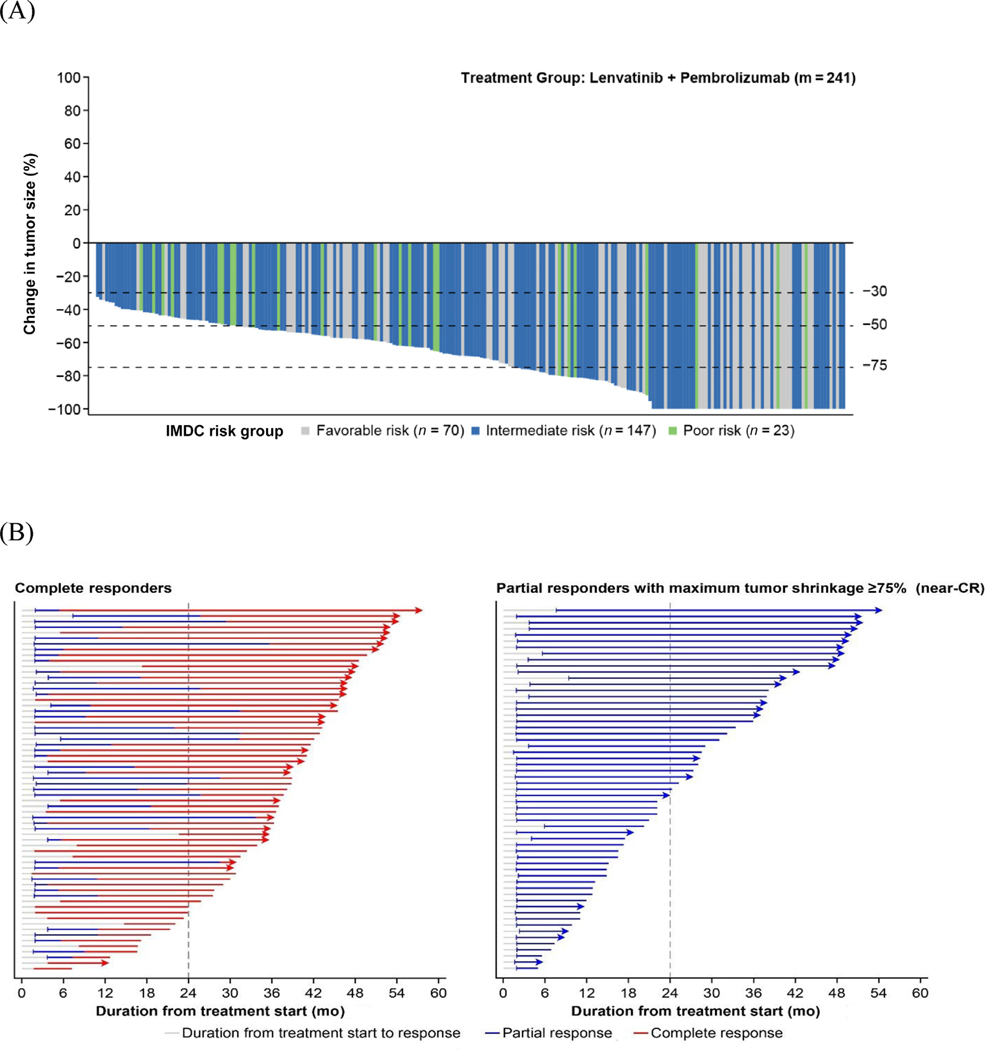

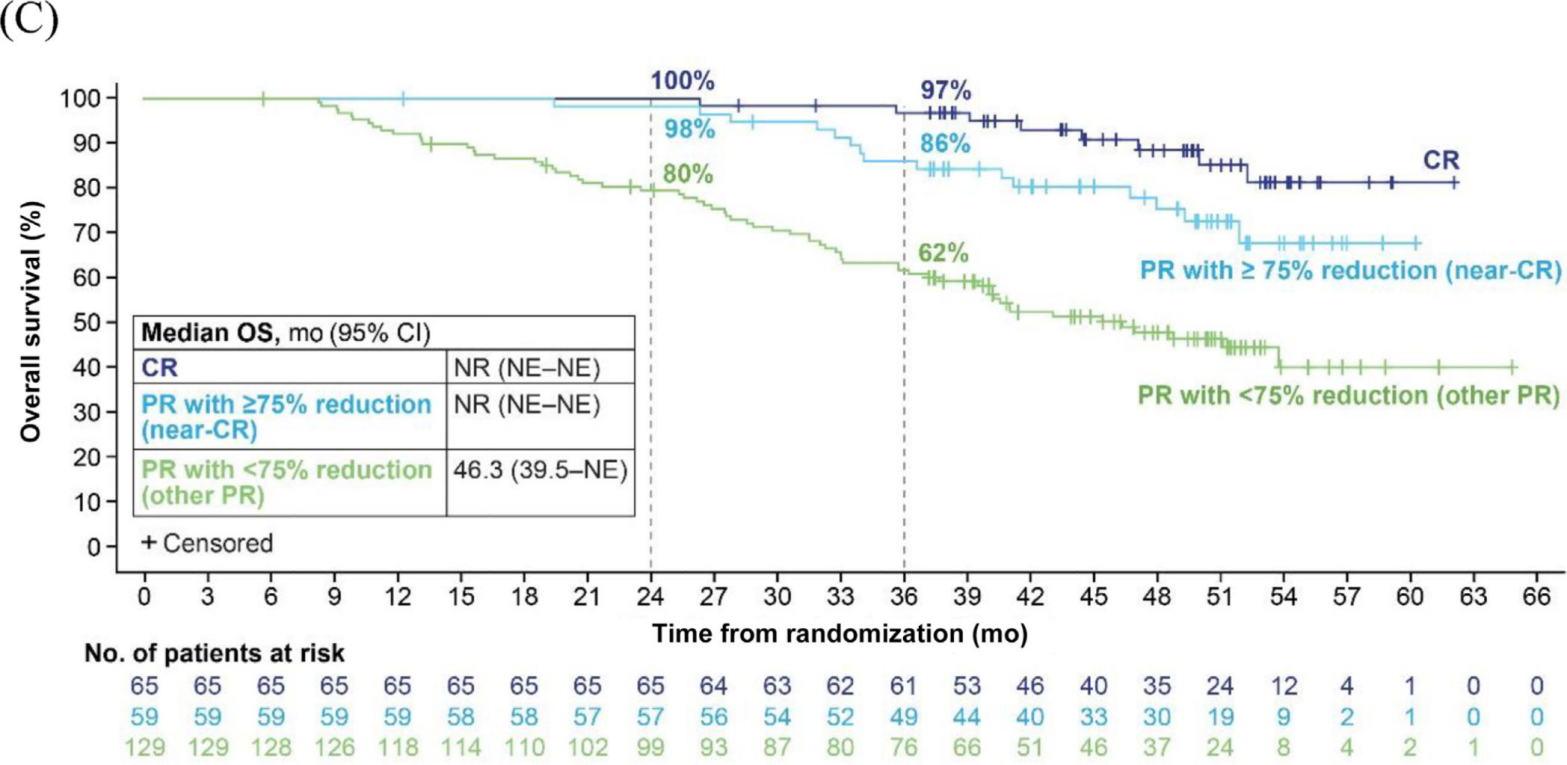
(A) Change in size of target lesions from baseline to postbaseline nadir by independent imaging review per RECIST v1.1 for responders per IMDC risk subgroups, (B) characterization of patients with a confirmed complete or near-complete response, and (C) OS by BOR category, all in the lenvatinib plus pembrolizumab arm of CLEAR. The IMDC prognostic group at baseline is based on total risk score from 6 prognostic factors at baseline: KPS, hemoglobin, corrected serum calcium, neutrophils, platelets, and time from first renal cell cancer diagnosis to randomization. IMDC risk groups were not a stratification factor and relevant data were derived programmatically. Figure 1A includes patients (m) with both baseline and ≥1 postbaseline target lesion assessment. In Figure 1B, arrows at the end of response lines indicate an ongoing response. The number of deaths in the responder subcategories were as follows: CR, 8 patients; near-CR, 15 patients; other PR, 65 patients. BOR, best overall response; CI, confidence interval; CR, complete response; IMDC, International Metastatic Renal Cell Carcinoma Database Consortium; KPS, Karnofsky Performance Status; NE, not estimable; NR, not reached; OS, overall survival; PR, partial response; RECIST v1.1, Response Evaluation Criteria In Solid Tumors version 1.1.

Among patients with an objective response in the L + P arm (*n* = 253), 65 had CR, 59 had near-CR, and 129 patients had other PR. Tumor responses were distributed across International Metastatic RCC Database Consortium (IMDC) risk groups (Table 1 and Fig. 1A). Of note, among the 65 patients with CR, 25 (38%) were classified with favorable IMDC risk, 37 (57%) with intermediate IMDC risk, and three (4.6%) with poor IMDC risk. Similarly, among the 59 patients who achieved near-CR, 20 (34%) had favorable risk, 34 (58%) had intermediate risk, and four (6.8%) had poor risk (Table 1).

DOR results for patients with CR or near-CR are shown in Figure 1B. Among patients with an objective response (CR + PR), the median DOR was 26.7 mo (95% CI 22.8–34.6) with L + P. The median DOR was highest for patients with CR (43.7 mo, 95% CI 39.2–not estimable [NE]), followed by patients with near-CR (30.5 mo, 95% CI 22.4–NE; Table 1). The probability of continued CR at 36 mo was 70% (95% CI 56–80%) with L + P.

Median OS was not reached for patients with CR or near-CR (Fig. 1C). For patients with CR, OS rates were 100% (95% CI 100–100%) at 24 mo and 97% (95% CI 88–99%) at 36 mo. For patients with a near-CR, the OS rates were 98% (95% CI 88–100%) at 24 mo and 86% (95% CI 74–93%) at 36 mo (Fig. 1C). For patients with other PR, median OS was 46.3 mo (95% CI 39.5–NE).

The median overall duration of treatment was 36.5 mo (interquartile range [IQR] 24.8–46.0) for patients with CR, 26.6 mo (IQR 17.3–41.8) for patients with near-CR, and 22.1 mo (IQR 12.4–35.6) for patients with other PR. At the data cutoff date, 21/65 patients with CR, 15/59 patients with near-CR, and 16/129 patients with other PR had ongoing lenvatinib treatment, while no patients had ongoing pembrolizumab treatment. Among 205 responders with dose reductions, the median time to first lenvatinib dose reduction was 3.12 mo (IQR 1.03–8.89) in the CR group (*n* = 56), 1.87 mo (IQR 0.72–5.29) in the near-CR group (*n* = 49), and 2.12 mo (IQR 0.89–5.19) in the other-PR group (*n* = 100). In the L + P arm, treatment was discontinued for 44 patients each with CR or near-CR, and for 113 patients with other PR. Among responders, L + P combination treatment was discontinued because of adverse events for ten patients with CR, 18 patients with near-CR, and 26 patients with other PR; L + P treatment was also discontinued because of radiological or clinical disease progression for 22 patients with CR, 20 patients with near-CR, and 68 patients with other PR. In addition, L + P treatment was discontinued because of patient choice, withdrawal of consent, or other reasons for 12 patients with CR, six patients with near-CR, and 19 patients with other PR. During survival follow-up, 24 patients with CR, 31 patients with near-CR, and 71 patients with other PR received any subsequent anticancer medication (Supplementary Table 1).

Previously published landmark analyses of the CLEAR trial at an earlier data cutoff date demonstrated the association of OS with tumor response [3]; however, the results presented here are descriptive in nature with no comparisons between the subcategories of responders within the L + P arm or to the comparator sunitinib arm.

In summary, the high and durable response rate is a notable feature observed with L + P treatment in the CLEAR trial. Responses were early, deep, and durable at long-term follow-up. Median OS was not reached for patients with either CR or near-CR.

Results from this long-term follow-up of CLEAR corroborate data from the primary analysis [1] and further support the use of L + P as a standard-of-care first-line treatment for patients with aRCC.

These data were presented in part at the 2023 Kidney Cancer Research Summit (Boston, MA, USA).

## Supplementary Material

1

2

## Figures and Tables

**Table 1 –:** Baseline characteristics of responders and summary of confirmed tumor responses (by Response Evaluation Criteria in Solid Tumors v1.1) by maximum tumor shrinkage from baseline among patients in the lenvatinib + pembrolizumab arm of CLEAR

Parameter	Response category		
	
	CR (*n* = 65)	Near-CR (PR with tumor shrinkage ≥75%) (*n* = 59)	Other PR (PR with tumor shrinkage <75%) (*n* = 129)
Median age, yr (IQR)	58 (47–65)	64 (57–71)	64 (57–71)
Male sex, *n* (%)	45 (69)	51 (86)	96 (74)
Race, *n* (%)			
White	48 (74)	43 (73)	102 (79)
Black or African American	0 (0)	0 (0)	2 (1.6)
Asian	16 (25)	14 (24)	23 (18)
Other	0 (0)	0 (0)	1 (0.80)
Data missing	1 (1.5)	2 (3.4)	1 (0.80)
Geographic region, *n* (%) ^a^			
Western Europe and North America	35 (54)	33 (56)	73 (57)
Rest of the world	30 (46)	26 (44)	56 (44)
Baseline KPS score, *n* (%)			
100–90	60 (92)	52 (88)	102 (79)
80–70	5 (7.7)	7 (12)	27 (21)
Lesion location, *n* (%) ^b,c^			
Lung	44 (68)	45 (76)	95 (74)
Lymph node	34 (52)	18 (31)	58 (45)
Bone	4 (6.2)	17 (29)	27 (21)
Kidney	4 (6.2)	11 (19)	48 (37)
Liver	5 (7.7)	10 (17)	20 (16)
Adrenal gland	8 (12)	10 (17)	19 (15)
Brain	0 (0)	1 (1.7)	3 (2.3)
Other	9 (14)	23 (39)	48 (37)
Metastatic organs/sites involved, *n* (%) ^c,d^			
0 organs/sites	0 (0)	0 (0)	5 (3.9)
1 organ/site	36 (55)	17 (29)	33 (26)
2 organs/sites	23 (35)	24 (41)	51 (40)
≥3 organs/sites	6 (9.2)	18 (31)	40 (31)
MSKCC prognostic group at baseline, *n* (%) ^a^			
Favorable risk	20 (31)	20 (34)	28 (22)
Intermediate risk	44 (68)	32 (54)	88 (68)
Poor risk	1 (1.5)	7 (12)	13 (10)
IMDC risk group at baseline, *n* (%) ^e,f^			
Favorable risk	25 (38)	20 (34)	31 (24)
Intermediate risk	37 (57)	34 (58)	82 (64)
Poor risk	3 (4.6)	4 (6.8)	16 (12)
Not evaluable	0 (0)	1 (1.7)	0 (0)
PD-L1 status, *n* (%) ^g^			
Positive (combined positive score ≥1)	26 (40)	18 (31)	35 (27)
Negative (combined positive score <1)	19 (29)	18 (31)	51 (40)
Data not available	20 (31)	23 (39)	43 (33)
Prior nephrectomy, *n* (%)	61 (94)	51 (86)	82 (64)
**Summary of tumor responses**			
Median time to first objective response, mo (IQR) ^h,j^	1.91 (1.9–3.7)	1.87 (1.8–2.0)	2.07 (1.9–4.6)
Median duration of objective response, mo (95% CI) ^i,j^	43.7 (39.2–NE)	30.5 (22.4–NE)	17.2 (12.5–21.4)
Duration of response, *n* (%)			
≥6 mo	64 (98)	54 (92)	101 (78)
≥12 mo	60 (92)	43 (73)	64 (50)
≥18 mo	55 (85)	35 (59)	45 (35)
Probability of continued response at 36 mo, % (95% CI) ^j^	70 (56–80)	40 (26–54)	23 (14–32)

CI = confidence interval; CR = complete response; IMDC = International Metastatic Renal Cell Carcinoma Database Consortium; KPS = Karnofsky performance status; MSKCC = Memorial Sloan Kettering Cancer Center; NE = not estimable; PD = progressive disease; PR = partial response; SD = standard deviation.

a Per interactive voice/response system.

b Patients may be represented in more than one category.

c Derived from information obtained from the independent imaging review.

d Kidney is not included in the number of metastatic organs/sites.

e The IMDC prognostic group at baseline is based on the total risk score for six prognostic factors at baseline: (1) KPS, (2) hemoglobin, (3) corrected serum calcium, (4) neutrophils, (5) platelets, and (6) time from first renal cell carcinoma diagnosis to randomization. IMDC risk groups were not a stratification factor and relevant data were derived programmatically.

f The overall objective response rate was 69% (76/110 patients; 95% CI 60–78%) in the IMDC favorable-risk subgroup, 73% (153/210 patients; 95% CI 67–79%) in the intermediate-risk subgroup, and 70% (23/33 patients; 95% CI 54–85%) in the poor-risk subgroup.

g PD-L1 status was determined using an investigational version of the PD-L1 immunohistochemistry 22C3 pharmDx assay (Agilent, Santa Clara, CA, USA) and a provisional combined positive score, defined as the number of PD-L1-staining cells (tumor cells, lymphocytes, macrophages) divided by the total number of viable tumor cells, multiplied by 100. The cutoff value is 1.

h Time to first objective response rate (mo) = (date of first objective response – date of randomization + 1) × 12/365.25 for patients with a best overall response of CR/PR.

i Duration of objective response (mo) = (date of progressive disease/death or censor date – date of first objective response + 1) × 12/365.25 for patients with a best overall response of CR/PR.

j Quartiles were estimated using the Kaplan-Meier method and 95% CIs using a generalized Brookmeyer and Crowley method.
