# Supplementary material for: Characterization of Responses to Lenvatinib plus Pembrolizumab in Patients with Advanced Renal Cell Carcinoma at the Final Prespecified Survival Analysis of the Phase 3 CLEAR Study
Source: Eur Urol. Author manuscript; Available in PMC 2025 Mar 9. (PMC11890201; doi:10.1016/j.eururo.2024.03.015)
Supplement: 1 [file NIHMS2049899-supplement-1.docx]

**Supplementary Table 1 – Summary of SSAs during survival follow-up for responders (Response Evaluation Criteria In Solid Tumors v1.1) using maximum TS to classify response**

| Category | Patients, *n* (%) ^a^ | | |
| --- | --- | --- | --- |
|  | CR | Near-CR (PR with  ≥75% TS) | Other PR (PR with  <75% TS) |
| Patients who started the study treatment | 65 (100) | 59 (100) | 129 (100) |
| Patients who discontinued the study treatment | 44 (68) | 44 (75) | 113 (88) |
| Patients who received any subsequent SSA during follow-up | 24 (37) | 31 (53) | 71 (55) |
| Anti-VEGF therapy | 19 (29) | 28 (47) | 68 (53) |
| PD-1/PD-L1 checkpoint inhibitor | 12 (18) | 6 (10) | 19 (15) |
| mTOR inhibitor | 2 (3.1) | 5 (8.5) | 4 (3.1) |
| CTLA-4 inhibitor | 3 (4.6) | 0 (0.0) | 6 (4.7) |
| Other | 4 (6.2) | 4 (6.8) | 7 (5.4) |

CR = complete response; PR = partial response; SSAs = systemic anticancer agents; TS = tumor shrinkage (percentage change from baseline in the sum of diameters for all target lesions).

^a^ Percentages are based on the total number of patients in the intent-to-treat population in the relevant treatment group. Patients with two or more anticancer medications may be counted in multiple categories.
