# Supplementary material for: Characterization of Responses to Lenvatinib plus Pembrolizumab in Patients with Advanced Renal Cell Carcinoma at the Final Prespecified Survival Analysis of the Phase 3 CLEAR Study
Source: Eur Urol. Author manuscript; Available in PMC 2025 Mar 9. (PMC11890201; doi:10.1016/j.eururo.2024.03.015)
Supplement: 2 [file NIHMS2049899-supplement-2.pdf]

Supplementary Appendix

Supplementary Table 1. Summary of Anticancer Medications during Survival Follow-Up for Responders and Maximum Percentage Change from Baseline in the Sum of all Target Lesion Diameters (RECIST v1.1)

	**CR (n=65)**	**Near-CR (PR with >=75% Tumor Shrinkage) (n=59)**	**Other PR (PR with <75% Tumor Shrinkage) (n=129)**
Patients who started study treatment, n (%)	65 (100)	59 (100)	129 (100)
Patients who discontinued study treatment	44 (68)	44 (75)	113 (88)
Patients received any subsequent systemic anticancer medication during survival follow-up by type	24 (37)	31 (53)	71 (55)
Anti-VEGF Therapy	19 (29)	28 (47)	68 (53)
PD-1/PD-L1 Checkpoint Inhibitor	12 (18)	6 (10)	19 (15)
MTOR Inhibitor	2 (3.1)	5 (8.5)	4 (3.1)
CTLA-4 Inhibitor	3 (4.6)	0 (0.0)	6 (4.7)
Other	4 (6.2)	4 (6.8)	7 (5.4)

Percentages are based on the total number of patients in the intent-to-treat population within the relevant treatment group. Patients with 2 or more anticancer medications may be counted in multiple categories.

CR, complete response; PD-1/PD-L1, programmed cell death-1/programmed cell death ligand 1; PR, partial response; RECIST v1.1, Response Evaluation Criteria In Solid Tumors version 1.1; VEGF, vascular endothelial growth factor.
